# Structured exercise programs for higher education students experiencing mental health challenges: background, significance, and implementation

**DOI:** 10.3389/fpubh.2023.1104918

**Published:** 2023-04-25

**Authors:** Ivan Jeftic, Bonnie J. Furzer, James A. Dimmock, Kemi Wright, Conor Boyd, Timothy Budden, Michael Rosenberg, Ben Kramer, Brett Buist, Ian Fitzpatrick, Catherine Sabiston, Melissa de Jonge, Ben Jackson

**Affiliations:** ^1^School of Human Sciences (Exercise and Sport Science), The University of Western Australia, Perth, WA, Australia; ^2^Telethon Kids Institute, Perth, WA, Australia; ^3^Department of Psychology, College of Healthcare Sciences, James Cook University, Townsville, QLD, Australia; ^4^Department of Exercise Physiology, School of Health Sciences, Faculty of Medicine & Health, University of New South Wales, Sydney, NSW, Australia; ^5^Faculty of Kinesiology and Physical Education, University of Toronto, Toronto, ON, Canada

**Keywords:** college, post-secondary, physical activity, depression, anxiety, substance use

## Abstract

The incidence of mental illness is greatest among young adults, and those enrolled in higher education may be particularly vulnerable compared to the general young adult population. Many higher education institutions employ student support staff tasked with implementing strategies to improve student wellbeing and mental illness. However, these strategies tend to be focused on clinical therapies and pharmacological interventions with limited lifestyle approaches. Exercise is an effective method for addressing mental illness and promoting wellbeing, yet widespread provision of structured exercise services to support treatment options for students with mental health challenges has not been fully realized. In an effort to guide exercise strategies for student mental health, we synthesize considerations for developing and delivering exercise programs in higher education settings. We draw directly from the evidence base on existing exercise programs in higher education; and the broader behavior change, exercise adherence, health psychology, implementation science, and exercise prescription literatures. Our broad considerations cover issues regarding program engagement and behavior change, exercise ‘dose’ and prescription, integration with other on-campus services, and robust research and evaluation. These considerations may provide impetus for widespread program development and implementation, as well as informing research focused on protecting and improving student mental health.

## 1. Introduction

Mental illness encompasses a range of conditions that adversely affect a person’s psychological state, and is a leading cause of disability (1). There is evidence to show that, relative to other population groups, the incidence of mental illness—including, for example, anxiety, depression, and substance use disorder—is greatest among young adults (2, 3). In Australia, the prevalence of anxiety disorders among young adults (aged 18–24 years) is 15.4%, and rates of affective and substance use disorders are 6.3 and 12.7%, respectively (2). Similarly high rates of mental illness are evident among young adults across the Western world, including, for instance, the UK (4), US (3), and Canada (5). Within the young adult population, it appears that students enrolled in tertiary or higher education (e.g., at a college or university) may be particularly vulnerable to mental illness compared to the general young adult population (6, 7). Researchers have shown that tertiary education students experience specific risk (i.e., health and social) factors that might negatively impact their mental health [e.g., Eisenberg et al., 2018 (8)]. These risks include substance use (particularly binge drinking and marijuana use), sleep problems, a lack of physical activity, experiencing assault or abuse, and financial stress (9).

Experiencing mental illness as a young adult is associated with negative short- and longer-term health outcomes. Short-term outcomes can include memory problems, increased perceptions of loneliness, and increased levels of fatigue (10, 11). Longer-term outcomes can encompass persistent emotional and physical health problems (12), labor market marginalization (13), and relationship dysfunction (14). Additionally, adverse outcomes may arise from mental illness that are specific to student populations—including to one’s academic performance and engagement (e.g., grades, attendance), and overall university experience (e.g., social isolation). For example, Bruffaerts et al. (2018) demonstrated that internalizing (e.g., depression, anxiety) and externalizing (e.g., inattentiveness, hyperactivity) mental health problems are associated with reduced academic functioning (15). This reduced academic functioning for students with mental health problems may be a result of lowered attendance and difficulties coping with their academic load (16). In addition, Salzer (2012) reported that, relative to those students who were not experiencing mental illness, those who were experiencing mental illness displayed lower engagement and poorer relationships on campus—both factors that are associated with lower graduation rates (17). Indeed, there is evidence that students with mental health problems display an increased rate of attrition from their studies altogether (18). Efforts are needed to curb the experiences and effects of mental illness among students.

A large proportion (i.e., over one third) of young adults in Australia (7), the UK (19), the European Union (20), North America (21), and Asia [e.g., Japan (22)] are enrolled in tertiary or higher education. These institutions, such as universities, act as a central ‘hub’ for a significant proportion of students’ daily activities (e.g., study location and services, health care services, recreational facilities), and, therefore, represent an important context for the provision of mental health support. Universities typically employ a variety of staff who can provide students with mental health treatment and support, both in-person and virtually (i.e., online). Qualified mental health support staff (e.g., counselors, psychologists, medical professionals) can assist by providing direct care and treatment, and/or liaising with other on- and off-campus support (e.g., psychological therapies, pharmacotherapy treatment). Moreover, university-based administrative and academic (i.e., non-clinical) staff who are educated in available campus supports can have a positive impact on student mental health (23). There are also additional university- or campus-based strategies designed to improve student ‘wellbeing’—including involvement in social activities and group skill-building sessions (24), assistance with gender or sexual orientation services (25), and the provision of sport, exercise, and recreation opportunities (26). In this paper, we focus our attention on the use of structured exercise programming as an on-campus ‘complement’ to psychological and other medical or clinical services. There is evidence that regular exercise can be beneficial in treating mental illness [e.g., Sancassiani et al., 2018 (27)], and universities are typically well-positioned to deliver such programming. However, as we outline in the following sections, greater efforts are needed to develop, resource, and integrate exercise programming on campuses as an adjunct treatment for students experiencing mental illness.

## 2. Exercise, mental health, and wellbeing

The effectiveness of exercise as a method to protect or improve mental health has been demonstrated in various populations and settings (27–29). In their systematic review, Stonerock et al. (30) concluded that exercise is an acceptable adjunct strategy for the treatment of anxiety for a population over the age of 18. Exercise significantly reduces anxiety when compared to a placebo or waitlist control (31). Moreover, the anxiolytic effects of exercise may also support mental health improvement when combined with cognitive behavioral therapy (32, 33). Exercise has also been recommended as an adjunct treatment for depression (34, 35). As a treatment for major depressive disorder, exercise alone has shown similar effectiveness to pharmacotherapy in terms of remission rates (36), and it has been identified that the combination of exercise and pharmacotherapy may be more effective in terms of symptomatology improvement than pharmacotherapy alone (37). Aside from anxiety and depression, exercise participation may also offer mental health benefits for those with a substance use disorder (38), psychosis (39), bipolar (40), posttraumatic stress disorder (41), and schizophrenia (42). There are multiple psychobiological mechanisms that may explain the relationship between exercise stimuli and positive mental health—including psychoendocrinological mechanisms, cardiovascular implication models, mechanisms enhancing neuroplasticity, and neurocognitive mechanisms outcomes [for more information see: Acevedo (43), Ekkekasis (44), or Smith and Merwin (45)]. The proposed mechanisms and models can aid in our understanding of how to best support a student population.

Exercise has also been shown to provide support for mental health and lifestyle behaviors within student populations specifically. For instance, researchers have found (a) a significant negative relationship between physical activity and perceived stress (46), (b) that moderate-to-high level intensity physical activity is associated with better sleep quality (47), (c) that participating in tennis exercise once weekly decreases depression and anxiety symptoms, and enhances wellbeing (48), and (d) that access to a recreation center is suggested to contribute to a healthy campus climate [see Jaworska et al., 2016 (49)]. Despite the benefits of regular exercise participation, it has been documented that students experiencing mental health problems display lower exercise participation levels than their peers who do not have mental health problems (8). Therefore, in order to provide the most comprehensive service and support for students who are experiencing mental health difficulties, it appears important that universities and other higher education institutions provide structured exercise opportunities for unique use and alongside primary clinical treatment approaches such as pharmacotherapy, psychological assessment and services, and counseling. Service provision of this kind is important in part because students value choice regarding treatment approaches and because they often desire alternatives to psychotherapy or medication—including lifestyle approaches such as exercise and diet (50, 51).

There are very few structured exercise programs operating on university campuses with the goal of supporting student mental wellbeing and complementing primary treatment options. We believe that only a handful of such programs have been described in the contemporary literature. deJonge et al. (52) analyzed the effectiveness of *MoveU.HappyU*—a University of Toronto physical activity and behavior change program for students who are not meeting physical activity guidelines and who are experiencing mental health challenges. *MoveU.HappyU* is a 6-week, one-on-one supervised exercise program, with each supervised session lasting one hour and autonomous exercise suggested up to 150 min per week. In their feasibility study, deJonge and colleagues presented preliminary evidence that the intervention positively affects students’ mental wellbeing. A modified version of the *MoveU.HappyU* program was established at the University of Windsor, a mid-sized Canadian University. The program *UWorkItOut Uwin* is a 6-week exercise training and counseling intervention (53). In their feasibility work, Muir and colleagues reported significant decreases in students’ anxiety and depression scores from pre-to post-intervention (53). A final intervention named *Western Wellcat* based at a U.S. west coast university [see Keeler et al. (54)] is a peer-led need-supportive physical activity intervention for students who are not currently physically active and who are experiencing mild-to-moderate depression. The intervention lasts between 8 to 10 weeks, with participants completing two 60-min sessions each week. In their report, Keeler et al. (54) concluded that the intervention group displayed improvements in depression and distress scores relative to the control group. The approach in these three programs differed in terms of length, implementation plans (e.g., type of exercise, inclusion of other behavioral therapies), and eligibility requirements, with the first two programs accepting students with any mental health challenge who are not meeting physical activity guidelines and the final program accepting students experiencing mild-to-moderate depression. Regardless of these differences, though, all studies found evidence of positive effects of exercise on mental health.

Despite compelling arguments for the role of structured on-campus exercise programs, there is little evidence to directly inform optimal program design and delivery. As such, it is important (and timely) to offer best practice principles that could be used to underpin on-campus exercise and mental health programs. Therefore, we present a series of theory- and research-informed considerations for the development and delivery of exercise-as-treatment programs for mental illness and psychological distress in tertiary education settings. In presenting these considerations, we were guided by two key principles—first, that structured exercise participation is an important adjunct treatment approach for mental illness among students and student-aged populations; and second, that exercise appears to be overlooked by many tertiary institutions in terms of their mental illness treatment plans. These considerations are presented as a summary of contemporary literature in the fields of psychology, exercise physiology and prescription, behavior change, and implementation science. We also studied literature regarding the development of on-campus programs of this kind, and consulted with ‘consumers’ in the form of discussions with participants in and deliverers of two such programs (i.e., *MoveU.HappyU* and a similar program that is currently being trialled at The University of Western Australia).

## 3. Program considerations

Outlined below are four broad considerations intended to inform the development of on-campus exercise programs to support student mental health treatment services (see Table 1). The four broad considerations below are not intended to encompass *all* the idiosyncrasies associated with on-campus exercise programs but are designed to provide programmers with a framework to tailor their unique programs to their institution and facilities.

**Table 1:** A summary of considerations and specific recommendations.

Broad consideration	Specific recommendation	Example or additional detail
Elements that support program engagement and behavior change	Use of trained peer mentors	Undergraduate or postgraduate; potential for expert mentors available in institutions with exercise-related health courses
	Implement motivationally-supportive strategies	Provision of need-supportive instruction to encourage high quality motivation for exercise [see (55) for a comprehensive overview of practical recommendations]
	Cater for variety and novelty in exercise	Build novel and/or varied experiences into an exercise program to promote vitalizing effects; use nature (green exercise)
	Harness gamification, mobile technology, and music	Friendly exercise competition with a mentor, using real or virtual teams or groups to work together in pursuit of an exercise goal; consider appropriate mobile applications, smart watches, activity trackers, or online supplementary materials; use self-selected music before and/or during exercise
	Dedicate attention and resources to successful ‘transitions’	Include a specific and defined transition phase during the program to promote maintenance of exercise habits
The best exercise ‘Dose’?	Session length	Suggested minimum exercise session length should be 30 min, with improved outcomes from sessions 45 min to 1 h in duration
	Session frequency	Multiple exercise sessions per week; some sessions may be prescribed but not supervised
	Program length	10-week program length has been suggested, with effects shown from programs as short as 6-weeks; longer programs, where feasible, may support efficacy
	Training modality	Aerobic and anaerobic both show improvement in mental health outcomes; allows for choice in exercise modality
	Appropriate measurement protocols	Physical (e.g., body weight, BMI, cardiovascular indicators, strength), and mental (e.g., DASS-21, IDS, K10) indicators
Whole-of-campus integration in design and delivery	Applying co-design for campus sensitivity	Ensure engagement of key on-campus stakeholders (students, medical staff, research experts, student welfare) throughout design, delivery, and evaluation process
	Safety and wellbeing of mentees	Embed exercise service within campus safety protocols; ensure connectivity and responsiveness for those involved
	Safety and wellbeing of program providers	Clear reporting protocols (incidents, deterioration); provision of clinical supervision for mentors within program
Build the evidence base with thorough research and evaluation	Use of best-practice research and reporting strategies	Consider process evaluations (56); adherence to CONSORT guidelines (57, 58); integrate comprehensive feasibility studies
	Methodological considerations	Consider qualitative and quantitative designs; ecological momentary assessment to determine in-program effects; consult expert Recommendations for Implementing Change Project (59)

### 3.1. Consideration #1: elements that support program engagement and behavior change

The provision of a structured service offers routine and accountability for individuals who are ‘prescribed’ with exercise (60). Prescribed exercise (or, exercise prescription) refers to the development of a tailored exercise routine, specific to the holistic needs of an individual (e.g., goals, presentation, health outcomes), often administered by an allied health exercise-based specialist (e.g., exercise physiologist, physiotherapist, or physical trainer). In that sense, those seeking to support exercise participation for students with mental illness may be more likely to generate engagement and adherence through directly referring students to a structured service compared to solely connecting to, or raising awareness of, leisure-time exercise options (51). However, even with a structural scaffold in place to guide people’s exercise behavior, it remains important to be aware of challenges associated with (a) promoting continued engagement in any referral program, and (b) realizing the full potential of a service by encouraging exercise participation *outside and beyond* one’s involvement in the program. In the material that follows, we broadly consider ways that program designers and researchers might, at least in part, address these challenges by drawing from psychological and behavior change principles. In doing so (here and throughout), we aim to provide practical suggestions that aid the development and structure of such programs.

Our first consideration in this section focuses on the value of suitable, trained, and supportive *exercise mentors*—to support accountability during a program, to encourage engagement in the program, and to help build and reinforce exercise habits outside the program. Within exercise and other health promotion contexts, there are well-established benefits associated with the use of peer mentors [e.g., significant increases in exercise frequency; see Martin Ginis et al. (61)]. On many university and higher education campuses, there are likely to be suitable undergraduate or postgraduate candidates for peer mentoring roles. And, in some institutions, expert mentors may be sourced and trained through cohorts studying in the fields of exercise and health sciences, sport science or kinesiology, and other exercise-related health sciences. It is a requirement for many of the students in these exercise-related fields that they undertake work-integrated learning placements, and accruing experiences within a mental health-support service is typically a growing requirement in these activities (62). Ideally, these peer mentors would not only occupy a role as an expert prescriber and manager of a student’s exercise activities, but would also provide compassion and social support through the development of caring relationships with student ‘mentees’. In such instances, peer mentors may benefit by obtaining valuable practical experience and developing their confidence and competence working with clients experiencing mental health challenges (63). Indeed, industry experience outside of the exercise setting specifically has been shown to broadly assist in the development of students’ self-efficacy regarding their workplace competencies [e.g., Reddan (64)]. Similarly, student ‘mentees’ (i.e., those experiencing mental health problems) benefit from the ongoing and consistent guidance and support of their mentor (65), as well as potentially increasing their openness and ability to share their experiences by working with the mentor who is ‘close’ to them and able to empathize and build rapport [e.g., Mead et al. (66)].

A skilled peer mentor may also play a role in supporting our second broad consideration in this section—specifically that programs are designed and delivered in ways that foster *high levels and quality of motivation*. Self-determination theory [SDT (67)] is a framework for understanding human functioning in which it is proposed that individuals’ motivation for an activity may vary in strength (i.e., from low to high motivation) *and* quality (i.e., reflecting their ‘reason for doing’ something). Researchers have demonstrated, in exercise (68) and other related fields (69), that higher quality motivational profiles are associated with greater behavioral engagement and persistence, as well as other desirable outcomes (e.g., enjoyment, sense of purpose) that are key to mental health support (54). These higher quality motivational profiles are characterized by strong autonomous (relative to controlled) motives. Autonomous motives represent engagement in an activity because (a) one derives inherent enjoyment and interest from the activity, (b) one feels the activity aligns with their sense of self, and/or (c) the activity generates valued outcomes. These motives are considered more desirable than those that are more ‘controlled’ in nature—such as pursuing an activity because of external pressure, guilt, or a desire to obtain reward or avoid punishment.

There are established ways through which program leaders may support more autonomous motivational patterns. These methods are focused on creating environments that support individuals’ basic psychological needs—for autonomy (i.e., a sense of volition), competence (i.e., feeling capable in a pursuit), and relatedness (i.e., meaningful, close connections to others). For a comprehensive overview of practical need-support recommendations in exercise settings, see Ntoumanis et al. (55) Briefly though, methods to support autonomy within a structured exercise program for students experiencing mental health difficulties may include the provision of choice regarding exercise timing and modality, inviting questions and conversation, and the provision of strong rationales for any program recommendations. Competence-supportive techniques may include providing adequate challenge, positive reinforcement, recognition of progress, and structured goal setting and monitoring. Finally, relatedness-support may be facilitated directly using skilled mentors, intentional pair- or group-based exercise, and/or specific actions such as showing appreciation, listening and empathizing, taking an interest in the students’ (mental health, educational, and exercise) progress, and accommodating individual’s preferences.

Beyond the use of mentors and motivationally-informed program delivery, there are a number of other considerations and behavior change techniques that may support program engagement—including providing feedback on behavior, demonstration of behaviors, action planning with the client, and information about consequences [for a list of behavior change techniques, see Michie et al. (70)]. First, it is important to comment on the value of *exercise goals*. Traditionally, exercise goal setting recommendations have been focused on formulating specific and measurable actions (71). More recently, however, evidence has emerged for the benefits of ‘open’, or more loosely defined, goals (e.g., “I want to be more active”). In their systematic review and meta-analysis, McEwan et al. (72) reported that goal setting interventions are effective for promoting physical activity participation *regardless* of goal specificity. A subsequent program of research has demonstrated further support for the benefits of ‘open’ (i.e., non-specific or -measurable) exercise goals, particularly when individuals are in the early stages of learning or activity adoption [see Swann et al. (73)]. Additionally, these goals may be more likely to be ‘flexible’ in nature within a population experiencing mental illness. In addition to providing program participants with input into their exercise goals (i.e., autonomy-support), and providing positive feedback relating to goal attainment (i.e., competence-support), program leaders may also encourage participants to consider open alternatives to the more traditional prescriptive and specific goals (e.g., “I’m going to see how many steps I can reach,” or “…to see how many new activities I can try”). And, to assist participants in reaching these goals, mentors may engage in appropriate *planning* activities—focusing not only on how, when, with whom, and where activities will be performed, but also ‘if—then’ contingencies (e.g., “if I miss my session this week, then I’ll…”). For an overview of planning activities with respect to exercise participation, see Sniehotta et al. (74), or Gollwitzer et al. (75).

Other features that may benefit engagement in programs of this nature include the appropriate use of *variety and novelty* during structured sessions. There are well-established ‘vitalising’ effects associated with expecting and experiencing variety in exercise (76, 77), and these benefits may be particularly pronounced for those who are not regular (i.e., ‘routinised’) exercisers. Moreover, building novel and/or varied experiences into an exercise program may also support participant adherence [see Sylvester et al. (77)]. In a practical sense, a focus on variety in session content may also appeal to those participants who form open goals relating to ‘trying out’ different or new activities. On a separate theme, it has also been demonstrated—within what is often referred to as the ‘green exercise’ literature [e.g., Pretty et al. (78)]—that *exercising in nature* may promote exercise enjoyment, mood benefits, and self-efficacy [e.g., Lahart et al. (79)]. For example, exercising near, or in, water has been shown to reduce anxiety (80), which may be particularly desirable for those experiencing mental health difficulties. As such, harnessing the natural environment and exposing participants to parks, trees, water, and other natural features, may be a widely feasible program consideration (81).

There may also be engagement-related benefits associated with the use of gamification principles, mobile technology, and music. *Gamification* involves the application of general elements of game-play (e.g., competition, points, unlocking activities or levels) to various activities (82, 83). Strategies to accommodate gamification in exercise and mental health programs for students on campuses may include ‘friendly’ exercise competition with a mentor, the use of real or virtual teams or groups to work together in pursuit of an exercise goal (e.g., “your task is to collectively run 10 kilometers”), unlocking challenges at different stages in a program, and/or an activity-based points tally that results in program completion or advancements. With or without gamification, the use of *mobile technology* (e.g., activity trackers, smart watches, mobile applications) may also encourage engagement-related benefits (84). Mobile technology may promote physical activity participation and adherence [for an example of an application with game play, see Althoff et al. (85)], and may be used to help track exercise and provide feedback. Approaches to integrate mobile technology in this way should be driven by the provision of choice and training for any intended user, as well as being mindful of the potential (positive or negative) role of the technology in relation to the user’s mental health (86).

There is also an extensive literature demonstrating the benefits of using *music* in exercise [see Karageorghis and Priest (87)]. Music in exercise can be integrated prior to a session or activity to have stimulatory effects, especially in relation to high-intensity exercise (88). Asynchronous music can have both stimulatory and motivational effects depending on whether stimulatory or motivational music is chosen (89, 90). And, in-task synchronous music can assist in an individual’s ability to regulate exercise intensity (91). Researchers have also demonstrated mental health improvements through the use of music therapy [see Keen (92)]. Importantly, music may also provide program leaders with an opportunity to cater to participant autonomy through user selection of musical accompaniments (93), and could contribute indirectly to relatedness-building if music selection is used as an opportunity by mentors to explore musical interests.

It is also important to briefly highlight that a ‘successful’ program for students with mental health difficulties should also seek to encourage behavior change—that is, to stimulate positive exercise habits and behaviors outside and beyond the program itself. Many of the strategies outlined above are likely to enhance the probability of longer-term behavior change at the same time as building in-program engagement (e.g., promoting motivation, setting goals and making plans). Nevertheless, it is also desirable that programs of this nature also feature explicit *‘transition-focused’* content, stages, and strategies. An in-program transition phase prior to the ‘removal’ of structured programming (i.e., when a student completes the program) is key for implementing and retaining lifestyle change (94, 95). Such a phase may be targeted towards (a) identifying and overcoming barriers that clients may face outside of the program, (b) connecting ‘graduating’ participants to those who have previously completed the program and who may become exercise partners or groupmates, and (c) building ‘bridges’ to established community- or other campus-based exercise opportunities. Establishing this process before the end of the structured program may also enable exercise mentors to physically attend community-based sessions alongside participants and to actively transition them into such services or facilities.

### 3.2. Consideration #2: the best exercise ‘Dose’?

In addition to some of the environmental, interpersonal, and perceptual factors that may support engagement in an exercise program for students with mental health difficulties, it is also important to consider the exercise dose that is recommended to elicit meaningful change in mental health outcomes for these individuals. We now turn our attention to this issue and draw from the exercise prescription literature to inform our considerations. Exercise prescription includes a specific plan of physical fitness-related activities, often administered by a rehabilitation specialist (e.g., exercise physiologist, physiotherapist, physical trainer), and designed for a specific purpose. The goals of exercise prescription vary according to the patient or client’s needs and health status, and the intended or desired health outcome change (i.e., clinician goals). When working with students who are experiencing mental health problems, a primary goal is to improve their psychological wellbeing and mental health; secondary goals may also include increasing exercise participation levels, improving physical fitness indicators, improving physical health outcomes, and developing a sense of community and social support.

In comparison to exercise prescription for physical or movement-related goals (e.g., increasing range of motion in a joint or overall cardiovascular capacity), there can be more ambiguity regarding exercise prescription for a psychologically-framed goal (e.g., alleviating anxiety or stress symptoms). However, guidelines regarding the preferred exercise treatment plan for individuals with mental health challenges have been proposed. It has been recommended that, where feasible, a minimum of three exercise sessions are provided per week (96), and that in intensive programs up to five sessions per week may be provided (97). With respect to session length, it has been suggested that, at a minimum, exercise should be performed for 30 min, but it has also been reported that improved outcomes may be derived from sessions lasting between 45 min and one hour (97–99). And, in terms of program duration, periods of 4 to 12 weeks in length have been suggested (98, 99), with the possibility to achieve improved efficacy for client outcomes through longer (e.g., >12-week) programs (100). Accordingly, with recognition towards resources and time-restraints, an ideal approach for number of sessions, length of sessions, and program length may likely be *“the longer, the better,”* where session facilitators may supplement program sessions with other exercise programs available on-campus (e.g., social competitions).

Physical activity interventions for individuals experiencing mental health challenges have utilized a variety of training modalities, and there is evidence for the effectiveness of anaerobic (e.g., weight-based) and aerobic (e.g., cardiovascular) activities in improving mental health outcomes (101, 102). As such, it may be most desirable for program leaders to find a balance between the imposition of some best-practice program/session structural features (e.g., ‘prescription’ in a classic sense) and the provision of autonomy-supportive strategies. For example, it is likely to be beneficial to have predetermined structure in terms of session length and duration, and for program duration, but client engagement and motivation may be boosted if there is also scope for client input into specific activities and modality (e.g., selecting activity types and time of day, determining variety, selecting natural environments and music). Additionally, the inclusion of affect-based prescription (i.e., exercise that is pleasurable and derives pleasant affective experiences) as part of client input can also assist in engagement and adherence (103). It is also important here to recognize that financial, staffing, and facility restraints may restrict any given organization’s capacity to meet best-practice exercise prescription recommendations for this population. As such, although adhering to these recommendations may increase the likelihood of achieving desirable program outcomes, we do not seek to dissuade researchers and organizations from developing programs in instances when it is known that all of these recommendations cannot be met. Indeed, we adopt the guiding principle that “*some is better than none, and more is better than some*” [for recent evidence in support of simple messaging such as this around exercise prescription, see Jones et al. (104); Nobles et al. (105); and for policy-level support, see Brown et al. (106); US Department of Health and Human Services (107)].

One important aspect to consider within the broader issue of determining an adequate exercise ‘dose’ is the associated need to demonstrate (through assessment) the nature and magnitude of any exercise-induced changes in mental health indicators. As a result, when considering scheduling and other programming issues, program deliverers should be mindful to build in appropriate and sensitive health measurement protocols (e.g., at intake, mid-point, and completion of the program). These measurements are particularly key at the beginning of a program in order to understand a participant’s ‘baseline’ on relevant physiological assessments, health assessments, and mental health variables, and to inform appropriate exercise prescription [e.g., the Preference for and Tolerance of the Intensity of Exercise Questionnaire (108)]. Relevant physiological assessments may include cardiovascular fitness (e.g., cardiopulmonary exercise test, aerobic power index assessment) and strength (e.g., grip strength, three-repetition maximum test) testing (109). Health assessments may include indicators of metabolic health (e.g., body composition, blood pressure, cardiovascular function) (94). Meanwhile, psychological instruments that are sensitive to change and appropriate for this population may include the Depression, Anxiety, and Stress Scale [DASS-21 or DASS-42; Henry and Crawford (110)], the Inventory of Depressive Symptomology [IDS-Clinician, IDS-Self Report; or Quick-IDS, such as QIDS-Clinician, QIDS-Self Report; Rush et al. (111)], the Kessler Psychological Distress Scale [K10; Andrews and Slade (112)], the Patient Health Questionnaire (113), the Generalized Anxiety Disorder scale (114), and the Major Depressive Inventory (115). Clearly, building rapport and showing compassion are key qualities for any program coordinator who may be charged with obtaining these assessments—placing individuals through a battery of some or all of these tests is potentially demanding and may be confronting in some respects [e.g., weight assessment; Alimoradi et al. (116)]. That being the case, prior to administering these surveys or tests, it would be valuable to ‘get to know’ the client, perhaps through an informal open-ended discussion. Parenthetically, such a discussion may also support priorities relating to psychological need support, safety, and exercise prescription by way of providing information about the participant’s exercise history, preparedness, and preferences. The aforementioned assessments, and open-ended discussions, can also help the exercise mentor provide a more tailored ‘dose’ (e.g., time spent exercising within and outside of the program) of exercise for each individual.

### 3.3. Consideration #3: whole-of-campus integration in design and delivery

University and other higher education campuses are culturally and vocationally diverse environments, housing students, academic and administrative staff, and services and providers (e.g., medical facilities, social hubs, student body representation, sport and recreational clubs and facilities). As a result of this diversity within and across campuses, it is unlikely that there exists an effective ‘one size fits all’ approach to the development and delivery of exercise programs designed to support students experiencing mental health difficulties. Accordingly, in order to optimize program feasibility and effectiveness, and to appropriately address the needs of stakeholders and end users, a process of ‘co-design’ may be a desirable program feature. Co-design is an increasingly popular implementation method within health promotion settings (117, 118). Health promotion settings on campus are likely to include (for example) a medical center, psychological services, and sports and recreation services. Individuals within each of these settings should be involved with program design, alongside other student wellbeing initiatives that may be offered on-campus (e.g., interventions targeting stress, peer support, academic engagement, and other lifestyle behaviors such as sleep or alcohol intake).” Although specific co-design stages and recommendations vary between (the many) frameworks available in the literature, there are often consistent themes that pervade these models. Boyd et al. (119), for example, outlined six core elements in their co-design process, each of which involves discussion with stakeholders and end-users: (1) engage, (2) plan, (3) explore, (4) develop, (5) decide, and (6) change. Similarly, Eyles and colleagues (120) outline their six-step process involving the assessment of evidence and user needs, development of the intervention or service, and prototype and pilot testing with continuous feedback. Key to the conduct of co-design, therefore, are recommendations to meaningfully engage with relevant stakeholders, respond to this engagement, and to regularly repeat this process throughout the development and delivery (and optimization) of a health service or program.

In the case of an exercise program for students with mental health difficulties, initial co-design activities may focus on devising the scope of the program, searching relevant literature, and soliciting information—through focus groups or one-to-one discussion—from students, student bodies and representatives, medical personnel, and campus services relating to sport, recreation, disability [also see, in a related sense, ‘co-production’; Smith and Wightman (121)], minority groups, and student wellness. These groups should also be consulted regularly through any subsequent planning and development stage, with a focus on driving feasibility, acceptability, reach, inclusivity, and accounting for student experiences stemming from intersectionality considerations (122). It would also be valuable at this stage to seek input from diverse populations on campus regarding people’s experiences of any similar programs (previously or on other campuses or in other workplaces). This stage also provides an opportunity for groups to ensure the program provides connectedness to the cohort it represents, and that it reflects the university culture (e.g., through the name of the program). Importantly, in a robust co-design process, these consultation activities continue well beyond the preparatory phase of an intervention or program, and are maintained with the purpose of seeking feedback and continual improvement (123). When these co-design processes operate most effectively [e.g., Move with Recovery (124), Staying Strong Toolbox (125), or Girls Active program (126)], they increase the likelihood of developing a service that is built on previous successful programs, is tailored to the host institution, meets the needs of stakeholders and end users, and is integrated within and implemented alongside existing on-campus (e.g., medical, psychological, counseling, disability support, health and safety, and recreation) services.

While considering the importance of integrating exercise with existing on-campus services, it is necessary to comment on the value that a ‘connected’ exercise service brings in supporting student safety and wellbeing. First, by building input from experts in student welfare into a co-design process, program leaders may be more likely to avoid pitfalls with respect to student safety and wellbeing [e.g., ensuring adequate insurance, screening, referral pathways, and facilities; see, for example, Hetrick et al. (127)]. In addition, by embedding an exercise service alongside existing safety and wellness groups on campus, program leaders ensure greater connectivity and responsiveness for those involved [see, for example, Thompson et al. (128)]. The completion of mental health first aid courses,^1^ or equivalent, is a critically important training requirement for those involved in the delivery and management of such a program (129). Clearly, in developing a program that supports students who may be experiencing severe mental illness, suicide (and other critical incident) risk is an important consideration. These risks may be mitigated through the identification of suicidal ideation and harmful behaviors, and through well-documented contingency plans for people who display suicidal tendencies at any stage of program involvement.

With the purpose of ensuring participant safety and efficient reporting protocols (e.g., in the case of deterioration, missed exercise sessions, crises or critical incidents, or for onward referral), exercise mentors and program leaders should be closely connected to other medical, student welfare, and mental health professionals. And, a program structure should be implemented that allows for a clear reporting chain for any adverse incidents (e.g., through critical incidence reports passed onto the program coordinator). In the case of the pilot program of this nature currently underway at The University of Western Australia, for instance, a program advisory group with representatives from these groups meets regularly, and efficient bi-directional referral and reporting is possible between the exercise program, student medical center, student psychological services, and all other health and welfare programs on campus. Finally, it is important to note that the risk of harm within programs of this nature is not limited only to clients (e.g., through self-harm), but also extends to those in a support role (e.g., program coordinators, exercise mentors) by way of negative psychological impact, compassion fatigue, or distress (130). Any exercise mentors involved in the program should be guided with self-care strategies and plans, and provided with an opportunity for clinical supervision [e.g., meeting regularly with a professional to discuss casework and other professional issues in a structured way; Milne (131)]. A program lead, such as an Exercise Physiologist (132), would also insulate mentors and enable them to share client- or program-related concerns.

### 3.4. Consideration #4: build the evidence base with thorough research and evaluation

Our final consideration relates to the role of researchers (and research) in the development and optimization of such exercise programs. It is a recognized priority in health promotion settings that researchers provide robust evaluation evidence regarding program feasibility and effectiveness—such activity is valuable not least because it aids with increasing the effectiveness and pace of uptake of complex interventions (133). Given that the academic literature regarding on-campus exercise programs for students with mental health challenges is at a developmental stage, there are important gaps in our knowledge about the implementation and efficacy of such interventions. Broad considerations in this respect include the need to demonstrate the effects of different program structures, exercise ‘doses’, and delivery styles on primary (i.e., mental health symptomatology) and secondary (e.g., social support, academic engagement, flourishing) outcomes. Researchers seeking to demonstrate these effects through, for example, randomized controlled trials, would be encouraged to pre-register their efforts, integrate process evaluation protocols into the design of such work (56), and adhere to best-practice design, analysis, and reporting standards [i.e., Consolidated Standards of Reporting Trials; Schulz et al. (57); Grant et al. (134)]. Similarly, given that programs of this nature are yet to become commonplace on university and other higher education campuses, there is significant scope for research focused on assessing feasibility [see Eldridge et al. (58); and for an example of such work, deJonge et al. (135)], implementation issues [e.g., Glasgow et al. (136)], implementation-effectiveness hybrid questions [see Curran et al. (137); Landes et al. (138)], and important considerations regarding the sustainability, scale-up, and scale-out [for an overview and example, see Aarons et al. (139); Smith et al. (140)] of programs of this nature. Program of this nature also provide the opportunity for collaboration through the integration of researchers in various fields including behavioral science (e.g., those housed in population health, psychology, public health, medicine, kinesiology, etc.), physiology, and implementation science.

There are important recommendations within the implementation science literature that may also aid in the design and development of research relating to these programs. For example, the 73 implementation strategies outlined in the Expert Recommendations for Implementing Change Project may provide a platform that (a) helps inform the design of engaging and effective programs in this area (59), and (b) provides insight into evaluation targets for researchers and identifies methods for program optimization. Finally, it is worth briefly commenting on the potential design of such evaluation activity. Intuitively, many researchers often consider quantitative, pre-and-post, controlled designs when seeking to demonstrate effectiveness of a program or service. Notwithstanding the value of such work, it is also beneficial to develop research protocols that are more sensitive to change and that provide more in-depth insight into participants’ experiences in such programs. To achieve greater sensitivity to change, repeated measures assessments [e.g., ecological momentary assessments built around weekly session attendance; see, for example, Furzer et al. (141)] may provide insight into fluctuations in exercise experiences and mental health symptoms, as well as the inter-relations between those trajectories (142). And, to enable better insight into participants’ experiences, qualitative approaches may be best suited to provide researchers with an ability to understand how, when, and why these programs work (or do not work) at their best [see, for example, Budden et al. (143), More et al. (144), DeJonge et al. (135), or Ashdown-Franks et al. (52)].

## 4. Conclusion

Young adults experience significant mental health challenges during what is a key developmental period and a critical time for the diagnosis of mental illness. Many young adults are enrolled as students in tertiary or post-secondary educational institutions, and there is evidence that the prevalence of mental health difficulties may be heightened in this cohort. Aligned with, and likely a result of, the prevalence of mental health challenges for this population, there are also often lengthy wait-lists for consultation with mental health professionals. The efforts of researchers, clinicians, and mental health professionals should, therefore, be concentrated on finding ways to support students’ mental health and offer effective treatment pathways for mental illness. One such treatment option is the provision of structured exercise services; however, programs of this kind are not commonplace on higher education campuses. The few programs that are reported in the literature have demonstrated the feasibility and potential effectiveness of exercise services, but guidelines and robust research evidence to inform the development of such programs is currently lacking. Drawing from psychology, exercise physiology and prescription, behavior change, and implementation science literatures, we offered four broad considerations for the development, delivery, and evaluation of such programs. These considerations are not intended to be an exhaustive list of all necessary structural or delivery issues. We anticipate there will be several additional (e.g., administrative or idiosyncratic) considerations beyond the ones we present below for those seeking to develop interventions of this kind. However, we consider these issues important to be mindful of in a general sense. We hope that our work contributes to a more coordinated effort to share expertise and resources in this area, and to support more efficient program design. Ultimately, our goal is that work contributes toward a more concerted approach to exercise support services for higher education students, and aids student mental health, personal development, and academic engagement.

## Author contributions

IJ wrote the initial draft of the manuscript, organized co-authors editing, implemented changes, and assisted in the development of one of the existing programs mentioned in the manuscript. BF, JD, KW, CB, TB, MR, BK, BB, IF, CS, MJ, and BJ all read and provided detailed feedback on the manuscript. All authors have read and approved the final manuscript.

## Conflict of interest

The authors declare that the research was conducted in the absence of any commercial or financial relationships that could be construed as a potential conflict of interest.

## Publisher’s note

All claims expressed in this article are solely those of the authors and do not necessarily represent those of their affiliated organizations, or those of the publisher, the editors and the reviewers. Any product that may be evaluated in this article, or claim that may be made by its manufacturer, is not guaranteed or endorsed by the publisher.

## Footnotes

^1^ mhfa.com.au
